# The potential impact and cost of focusing HIV prevention on young women and men: A modeling analysis in western Kenya

**DOI:** 10.1371/journal.pone.0175447

**Published:** 2017-04-12

**Authors:** Ramzi A. Alsallaq, Jasmine Buttolph, Charles M. Cleland, Timothy Hallett, Irene Inwani, Kawango Agot, Ann E. Kurth

**Affiliations:** 1 New York University, New York, United States of America; 2 Imperial College London, London, United Kingdom; 3 University of Nairobi, Nairobi, Kenya; 4 Impact Research & Development Organization, Kisumu, Kenya; British Columbia Centre for Excellence in HIV/AIDS, CANADA

## Abstract

**Objective:**

We compared the impact and costs of HIV prevention strategies focusing on youth (15–24 year-old persons) versus on adults (15+ year-old persons), in a high-HIV burden context of a large generalized epidemic.

**Design:**

Compartmental age-structured mathematical model of HIV transmission in Nyanza, Kenya.

**Interventions:**

The interventions focused on youth were high coverage HIV testing (80% of youth), treatment at diagnosis (TasP, i.e., immediate start of antiretroviral therapy [ART]) and 10% increased condom usage for HIV-positive diagnosed youth, male circumcision for HIV-negative young men, pre-exposure prophylaxis (PrEP) for high-risk HIV-negative females (ages 20–24 years), and cash transfer for in-school HIV-negative girls (ages 15–19 years). Permutations of these were compared to adult-focused HIV testing coverage with condoms and TasP.

**Results:**

The youth-focused strategy with ART treatment at diagnosis and condom use without adding interventions for HIV-negative youth performed better than the adult-focused strategy with adult testing reaching 50–60% coverage and TasP/condoms. Over the long term, the youth-focused strategy approached the performance of 70% adult testing and TasP/condoms. When high coverage male circumcision also is added to the youth-focused strategy, the combined intervention outperformed the adult-focused strategy with 70% testing, for at least 35 years by averting 94,000 more infections, averting 5.0 million more disability-adjusted life years (DALYs), and saving US$46.0 million over this period. The addition of prevention interventions beyond circumcision to the youth-focused strategy would be more beneficial if HIV care costs are high, or when program delivery costs are relatively high for programs encompassing HIV testing coverage exceeding 70%, TasP and condoms to HIV-infected adults compared to combination prevention programs among youth.

**Conclusion:**

For at least the next three decades, focusing in high burden settings on high coverage HIV testing, ART treatment upon diagnosis, condoms and male circumcision among youth may outperform adult-focused ART treatment upon diagnosis programs, unless the adult testing coverage in these programs reaches very high levels (>70% of all adults reached) at similar program costs. Our results indicate the potential importance of age-targeting for HIV prevention in the current era of ‘test and start, ending AIDS’ goals to ameliorate the HIV epidemic globally.

## Introduction

The world has committed to ambitious targets to reach high levels of HIV testing and starting antiretroviral therapy (ART) upon HIV diagnosis to achieve viral suppression as well as to prevent HIV infections in the first place [1]. Whether the approaches to achieve these goals can be applied to equivalent effect among young people as well as adults is an open, and timely, question. Many youth are not currently able to access HIV testing, evidence-based prevention services, or ART [2–5]. Recent publication of the SEARCH study conducted in the high HIV-burden countries of Kenya and Uganda using a mobile multidisease approach found that while otherwise highly successful in getting adults to HIV test and start ART, and young people tested at higher levels than before [6], youth need further outreach for optimal HIV testing and treatment. Understanding not only geospatial “hotspots” in generalized HIV epidemics (western Kenya for example [7]) but subpopulations (such as youth) that are characterized by disproportionate rates of transmission can support better management of scarce resources allocated for HIV prevention and control [8].

Several lines of evidence indicate that young adults/youth, 15–24 years of age, are a key demographic to target with HIV prevention interventions. Ecological studies have indicated that sexual debut (including forced coitarche) at a young age is more common in sub-Saharan African countries with high HIV prevalence and generalized epidemics [9]. HIV seroprevalence surveys from these countries indicate that relatively high levels of HIV prevalence are persistent among youth cohorts, especially females [10,11]. Approximately 80% of youth living with HIV globally are in sub-Saharan Africa, encompassing primarily young females [12]; further, in generalized epidemics youth are exposed to HIV risk over their entire sexual lifespan.

It is well-established that administering ART to persons living with HIV reduces HIV-related mortality and improves clinical outcomes [13], thus serving as an effective secondary and tertiary clinical prevention strategy for people living with HIV themselves. ART also significantly reduces HIV transmission to HIV-negative partners: 'treatment as prevention' (TasP) supporting primary HIV prevention [14]. Recognizing this, the WHO has endorsed offering ART to all persons with HIV (regardless of CD4 level) [15]. These concepts of ART as primary to tertiary HIV prevention will be referred to throughout this paper as ‘TasP’. However, many countries have not fully adopted these recommendations into their national guidelines, and in many countries the decline in HIV incidence among youth has been lower than targeted previously in the World Health Assembly 2011 strategic goals [16].

Current estimates are that around 17 out of 35 million persons who are HIV infected are on ART, and that the fastest growing population with incident infections is youth. In the current context of limited resources for HIV epidemic management and optimal implementation strategies for TasP to prevent further infections, decision making is needed. Because typically in generalized epidemics the number of HIV-infected persons is distributed over all CD4 ranges, any TasP strategy must involve treatment at low CD4 counts. One possible approach is through prioritizing infected persons at early stages of sexual debut for ART following diagnosis (TasP) while continuing to provide ART for those presenting for care at lower CD4 count thresholds. Thus, it becomes important to compare age-focused TasP to approaches with a wider population focus. This paper describes mathematical modeling work to compare youth- vs. adult population focused approaches to combination HIV prevention to support HIV epidemic control goals.

In this study we assessed the potential impact and costs of combination HIV prevention for youth (15–24 years of age) compared to a limited number of interventions focused on adults (15+ years of age) in Nyanza, western Kenya using a deterministic compartmental mathematical model. Nyanza has an HIV prevalence of 15 percent and two-thirds of its population is under age 24 years [17]. The evidence-based interventions included HIV testing, risk reduction counseling following HIV diagnosis in the form of increased condom use [18,19], and TasP for HIV-diagnosed youth [14]. Additionally, we included interventions that reduced HIV-susceptibility for HIV-uninfected youth and we assumed these interventions were tailored to the young person’s age and sex/gender. These interventions included voluntary medical male circumcision (MC) for young men [20–22], pre-exposure prophylaxis (PrEP) for most-at-risk young women [23–26], and cash transfer (CT) to support school retention for in-school young girls [27,28]. We elucidated the potential of various permutations of coverage or efficacy levels of these interventions focused on youth, in comparison with adult-focused strategies encompassing increased adult HIV testing with TasP and condoms.

The modeling analyses in this study informed the selection and piloting of a scalable package of combination HIV prevention interventions in sub-Saharan African contexts tailored by age and gender, the MP3 Youth study (NIH R01AI094607, Kurth and Inwani PIs).

## Methods

### Model structure and parameterization

We developed a deterministic compartmental mathematical model in Matlab (Natick, Massachusetts, USA) for HIV transmission among heterosexuals in hyperendemic communities (see S1 File for model equations and Table 1 for model inputs). All used software programs are available by request from the corresponding author.

**Table 1:** Model inputs.

Parameter	Default Value	Other values in sensitivity analyses		Sources and Notes
		Different value 1	Different value 2	
Mean years of survival with untreated HIV by age at infection				
<5	3.3	80% of default values	120% of default values	[31,32]
5–24	14.0	80% of default values	120% of default values	[31,32]
25–29	13.0	80% of default values	120% of default values	[31,32]
30–34	11.0	80% of default values	120% of default values	[31,32]
35–39	10.0	80% of default values	120% of default values	[31,32]
40–44	9.0	80% of default values	120% of default values	[31,32]
45–49	7.0	80% of default values	120% of default values	[31,32]
50–54	6.0	80% of default values	120% of default values	[31,32]
≥55	5.0	80% of default values	120% of default values	[31,32]
HIV transmission probability per sexual act per stage of infection (no interventions)				
From chronic stage III	0.0008	–	–	[33]
From acute stage	0.0216	–	–	[33]
From late stage IV	0.0056	–	–	[33]
To females	0.0016	–	–	[34,35]
Behavior related parameters				
Assortativity in mixing by sexual risk	0.89 (Fitted)	0.1	0.5	Representative range
Assortativity in mixing by age	0.3 (Fitted)	0.1	0.9	Representative range
Maximum age difference between sexual partners (years)	10 (up to two age groups)	0 (same age group)	20 (up to four age groups)	[36]
Partner change rates relative to low risk category	9 for intermediate risk group and 117 for high risk group (Fitted)	50% of default value for both risk groups	150% of default value for both risk groups	Representative range
HIV treatment related parameters				
Infectivity while on HIV treatment	96% reduction from when not treated	80%	92%	[14,37]
Drop out from HIV treatment for youth	10% per person year	1%	20%	Representative range in similar settings [38]
Excess mortality while on HIV treatment compared to when not infected	5% and 8% per person year depending on the stage of infection in which treatment is initiated	80% of default value in both cases	120% of default value in both cases	[39]
Costs (2012 US Dollars or US$) and related parameters				
HIV testing (per test)	$12	–	–	Representative value consistent with values used in a recent study by the Modelling Consortium [40]
ARV drug (per person per year)	$168	–	–	From NASCOP and a cohort study [41,42]
HIV care (symptomatic HIV CD4<250)	$168	$500	$1000	Representative range for sub-Saharan Africa [40,41]
HIV care (diagnosed CD4≤250–350)	90% of symptomatic HIV care cost	–	–	Consistent with recent estimates from various African countries [40]
HIV care (diagnosed CD4≥350)	60% of symptomatic HIV care cost	–	–	Consistent with recent estimates from various African countries [40]
Circumcision (per circumcised male)	$43	–	–	From Kenya MC National Strategy [43]
Condoms (per condom)	$0.125	–	–	Assumed [63]
Cash transfer (per person per year)	$300 ($25 per month)	–	–	
PrEP (per person per year)	$200	–	–	Including service and drug costs
Annual discount rate	3%	1%	15%	Representative range [44]

We stratified the model by HIV status and stage, age, sex, and sexual risk behavior. To represent interventions, the model is further stratified by status of: HIV testing, HIV treatment/ART, male circumcision (MC), pre-exposure prophylaxis (PrEP) use, and cash transfer (CT) use. We represented heterogeneity in risk behavior by three groups differing in partner change rates and condom use. Rates of partner change for sexually active adults declined between the years 1998–2003, representing the reported declines in average number of partners noted in demographic surveys in Kenya [29,30].

**Fig 1:**
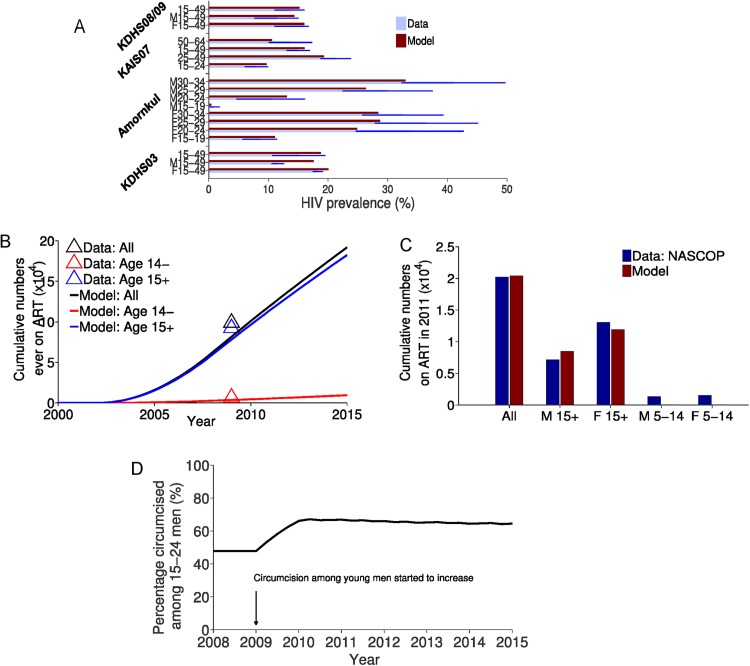
Calibration of model parameters using epidemiological and behavioral data from Nyanza. A) Calibration of model uncertain parameters using age-gender-specific prevalence data at different years from Kenya's KDHS 2003 and 2008/09 and KAIS 2007 surveys [17,53,54] and a published report by Amornkul et. al. [55]. Dark blue lines indicate confidence intervals of prevalence data. Prevalence values among males and females of various age intervals are indicated with M and F otherwise no indication for values among all adults of that age. Calibration to B) age-specific data on numbers ever initiated treatment up to 2009 as reported in NASCOP 2009 report [46], and to C) age-gender-specific data on numbers ever initiated treatment during 2011 from NASCOP [42]. Calibrations in B) and C) are used to adjust proportion initiating treatment at CD4 = 350 and at CD4≤250 cells/mm^3^ in fitted case (estimated at 10% and 90% of those with CD4≤350 cells/mm^3^, respectively). D) Assumed baseline male circumcision coverage among young men representing recent increase in proportion of young males (age 15–24) getting circumcised in Nyanza [46,54,56,57] (incorporated in the fitted case and all scenarios).

The natural history of untreated HIV was represented by six stages starting with the acute stage and ending with AIDS and differing by duration, CD4 count and infectivity [31,32,45]. Mean survival duration of untreated HIV infection was in the range of 3–14 years depending on age at infection (Table 1), as informed by Todd et al. analyses for low to middle income countries [31]. By calibrating model parameters to ART initiation data from Nyanza [42,46] (Fig 1B and 1C), we estimated that about 10% of persons who are infected and survive to CD4 count/mm3 of 350 initiate HIV treatment at CD4 count/mm3 of 350 while the remaining 90% initiate treatment late at CD4 count/mm3 of ≤ 250. With HIV diagnosis, in simulated testing campaigns, treatment could be initiated immediately following HIV diagnosis (depicted as TasP, which now forms the WHO 2015 guideline [47]) or promptly at CD4 count of 350 for tested persons with ages 25 years or more. In the model, persons on ART have an onward transmission probability that is 96% less than untreated persons in the chronic stages of HIV [14].

Nyanza’s population size was obtained from census data [48,49]. Age-specific fertility and mortality rates were from the 1998 Kenya's demographic survey [50] before HIV substantially affected those demographic rates. We assumed a 10 year maximum age difference in heterosexual partnerships between different age partners with men always being the older partners except for school-aged men (age 15–19) who form partnerships with girls of the same age as indicated in age mixing observational studies from Africa [36,51,52]. Age-specific HIV prevalence data from three population surveys and from one published report [17,53–55], cumulative numbers on ART in Nyanza from one population survey [46] and from personal communication with Kenya’s National AIDS & STI Council (NASCOP) [42], and the recent increase in circumcision prevalence among young men in Nyanza reaching 67% from national surveys [46,54,56,57] were used to calibrate model parameters and get the fitted case (Fig 1) (for details see S1 File).

ART treatment is assumed to increase survival and reduce infectivity [14,37] and persons on treatment were modeled as dropping out at a 10% rate per person per year, a representative treatment attrition level for Nyanza [38]. We allowed re-initiating of ART by dropouts who previously initiated treatment at CD4≥250 cells/mm^3^. A 40% HIV testing coverage level among 15–49 year old adults from national surveys [17] was assumed at baseline and we tracked the number of persons knowing their HIV status (“HIV diagnosed”).

### Model implementation of interventions

#### HIV testing and assumed services for HIV-diagnosed persons

From 2014, we assumed all interventions flow from HIV testing as the portal or entry point. For HIV testing, we studied two testing scenarios differing by age group. The first was adult testing (ages 15 or older), with coverage of 40%, 50%, 60%, 70% and 80% among adults of ages 15 and up; reached in one year and maintained in the future. The 40% level was informed by recent national surveys for persons in Kenya that had a HIV test in the previous year [56]. The second scenario was youth testing (focusing specifically on ages 15–24) with 80% coverage among youth reached in one year and maintained in the future, while status quo testing levels among other age groups were assumed.

We assumed an increase in condom use among persons who are diagnosed with HIV in the simulated testing campaigns. It is assumed that those persons reduce the number of unprotected sexual acts by 10% through the use of condoms in sexual acts with a sexual partner of discordant or unknown HIV status for a mean duration of 4 years, and revert back to baseline levels after that (see Table D in S1 File for baseline levels of condom use [56]).

#### Standard scenario

All scenarios are assumed to be incremental to a scenario with 67% MC coverage among young men, 40% testing coverage among adults, and prompt ART initiation at CD4 count 350 (Table 2). However, we also compare performance of scenarios to a standard scenario of 67% MC coverage among young men, 40% testing coverage among adults with immediate care and TasP i.e. ART following diagnosis with HIV regardless of CD4 count (Standard, Table 2).

**Table 2:** Assumptions of first set of comparison scenarios.

Assumption	Fitted case	Standard	Adult testing & TasP/condoms	Youth 80% testing & TasP/condoms
Coverage cap among adults reached in one year unless otherwise indicated (proportion ever tested)	40%	40%	40%,50%,60%,70% or 80%	coverage reaches 78% over 20 years
Coverage cap among youth reached in one year unless otherwise indicated (proportion ever tested)	coverage reaches 31% over 20 years	coverage reaches 31% over 20 years	coverage reaches 31%-68% over 20 years	80%
Reduction in the number of unprotected sexual acts through the use of condoms following HIV-diagnosis	0%	0%	10%	10%
Percentage of persons reducing unprotected sexual acts through the use of condoms following HIV diagnosis	0%	0%	100%	100%
Mean number of years over which the reduction of unprotected sexual acts lasts after HIV diagnosis	─	4	4	4
Efficacy of condoms	80% [58,59]	80% [58,59]	80% [58,59]	80% [58,59]
Percentage of diagnosed persons initiating TasP	0%	100%	100%	100%, if young
Percentage of diagnosed persons initiating ART at CD4≤350 cells/mm^3^	Not based on diagnosis	100%	100%	100% if not young
Percentage of infected persons who have never been diagnosed by the HIV testing campaigns with CD4≤350 cells/mm^3^ initiating ART at CD4 = 350 cells/mm^3^	10%	10% (as in fitted case)	10% (as in fitted case)	10% (as in fitted case)
Percentage of infected persons who have never been diagnosed by the HIV testing campaigns with CD4≤350 cells/mm^3^ initiating treatment late at CD4≤250 cells/mm^3^	90%	90% (as in fitted case)	90% (as in fitted case)	90% (as in fitted case)
Efficacy of treatment in reducing onward HIV transmission	96% [14]	96% [14]	96% [14]	96% [14]
Percentage of uncircumcised young males getting circumcised irrespective to HIV status	37%	37% (as in fitted case)	37% (as in fitted case)	37% (as in fitted case)
Circumcision uptake among older males	0%	0%	0%	0%
Efficacy of circumcision in reducing susceptibility to HIV acquisition among men	60% [20–22]	60% [20–22]	60% [20–22]	60% [20–22]

In our first set of comparisons (Table 2), we studied the potential of youth-focused HIV treatment at diagnosis and condom use in comparison with scenarios of increased HIV testing among adults with treatment at diagnosis and condoms.

#### Youth-focused interventions reducing susceptibility to HIV

In the second set of comparisons (Table 3) we considered increased male circumcision (MC) uptake among HIV-negative young men beyond current levels in Nyanza [56,60], and we excluded HIV-positive young men in these further circumcision efforts as little evidence exists that circumcising HIV-infected men reduces onward transmission [61]. In this model, 37% (baseline level), 50%, or 80% of uncircumcised HIV-negative young males are circumcised following HIV testing.

**Table 3:** Assumptions of second set of comparison scenarios.

Interventions for HIV-susceptible youth in context of youth 80% testing & TasP/condoms			
MC	CT	PrEP	
Proportion of young men (age 15–24) getting circumcised following HIV-negative test	Proportion of in-school girls (age 15–19) who are on CT following HIV-negative test	Proportion of 20–24 year-old women getting PrEP following HIV-negative test	Efficacy of PrEP of reducing HIV susceptibility
37% (baseline), 50%, or 80%	0% (baseline), 20%, 50%, or 90%	0% (baseline), 6%	0%, 30%, 60%, or 90%

We specified the age group among young females with the highest HIV prevalence and number of partners (20–24 year old females [17]) for the oral PrEP intervention. From survey data [17], we estimated that up to 6% of 20–24 year-old women in Nyanza are eligible to take PrEP based on the two criteria that they engage in high risk sexual acts and are able to take PrEP (gender-age-risk-tailored PrEP). We studied efficacy levels of 0%, 30%, 60% and 90% for the efficacy of PrEP in reducing HIV susceptibility among young women [23,24,26]. In our model, we assumed that girls on PrEP stop it immediately if they are infected and diagnosed, otherwise they stop at age 25.

The eligible proportion in Nyanza of school-aged young girls (ages 15–19) for cash transfer (CT) defined by being school attendant is 20% [17] (gender-age-school-tailored CT). Following findings from cash transfer studies in Tanzania, Malawi, and Kenya [27,28,62,63], we assumed that CT indirectly limits girl’s sexual mixing with 20+ year-old men, such that the proportion of girls recruited to CT represents the percentage reduction in girls’ partnerships with 20+ year-old men. We studied coverage levels of 0%, 20%, 50%, or 90% for CT among in-school 15–19 year-old HIV-uninfected girls following HIV testing. The maximum fraction of 90% is the proportion found to receive significant gifts from older men in one study [28]. In our model, we assumed that girls receiving CT stop receiving CT upon HIV diagnosis in the simulated HIV testing campaigns; otherwise, they stop at age 20. In the scenarios when CT is provided with TasP and condoms, those young girls who are diagnosed with HIV are assumed to be linked immediately to HIV care and treatment (TasP) as well as given condoms to use with their HIV-negative partners.

We studied various levels of uptake and efficacy of MC, PrEP and CT interventions among HIV-susceptible youth. The total number of combinations formed was 3x4x5 = 60 scenarios; including the scenario of youth 80% testing & TasP/condoms with no further gender-age-tailored interventions and excluding three redundant scenarios of 0% PrEP coverage.

#### Cost assumptions

We conducted our economic assessment for cost-effectiveness from a health provider perspective accounting for all costs in 2012 United States Dollar amounts (US$) and outcomes of interventions over time. We discounted costs and outcomes at 3% annually. Table 1 lists our cost assumptions. We assumed HIV testing costs $12 per person tested within the range of pooled cost in a recent study [40]. The annual antiretroviral drugs (ARVs) (using TDF/3TC/EFV) cost of $168 per person per year was obtained from personal communication with NASCOP.

For later stages of HIV infection (CD4≤250 cells/mm^3^), the per year HIV care costs (that is, costs of services other than ARV medications) are set equal to ARV costs, based on a recent micro-costing analysis for provision of treatment at three rural sites in Kenya in 2009 including one in Nyanza [41] with costs accounting for all resources used by the provider. Care costs at a given HIV-stage are assumed to be equal for in-care persons irrespective of whether they initiated treatment at that stage (on treatment), in pre-treatment stage, or dropped out of treatment assuming decreasing costs for earlier HIV stages. We assumed 90% and 60% of care costs in late stages of infections for persons with 250<CD4≤350 and CD4>350 cells/mm^3^; respectively. We vary the assumption on care costs in our analyses.

The cost of MC was $43 per male circumcised [43]. We estimated condoms to cost $0.125 per condom and CT to cost $300 per eligible female per year ($25 per month incentive), while we set PrEP costs at $200 per eligible female per year. Weights for DALYs were imported from the Global Burden of Disease Study [64]. To acknowledge the worsening health of persons dropping out of treatment due to increased risk of virologic failure among those with poor adherence to ART [65], we assumed a disability weight for dropouts equal to the weight for untreated stage of HIV when CD4 is between 250 and 350 cells/mm^3^. We did not incorporate separate program costs or costs averted from preventing new HIV infection.

### Analyses outline (Fig 2)

**Fig 2:**
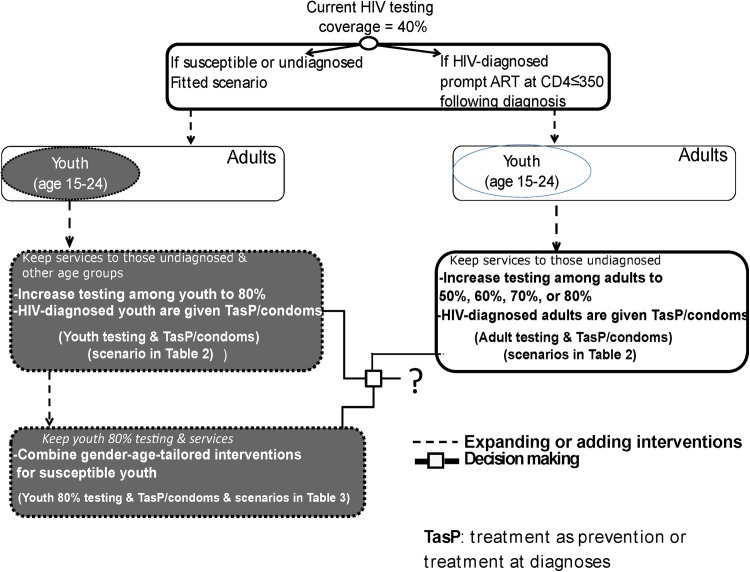
A schematic outlining our analysis. Comparison scenarios depicted in the three bottom boxes connected by the decision making solid lines are incremental to the scenario shown in the top box of: 1) 40% HIV testing among adults ages≥15 and prompt initiation of ART at CD4 ≤350 cells/mm^3^ 2) undiagnosed infected persons presenting late to care/initiating treatment at CD4 ≤250 cells/mm^3^, and 3) male circumcision uptake of 37%. The comparison scenarios focusing on adults consisted of increased HIV testing among adults (from 40% up to 80% coverage) and provision of TasP and condoms. The rest of the comparison scenarios depicted in shaded blocks focus on youth where in one set HIV testing coverage is increased specifically among youth to 80% and diagnosed youth are provided TasP while condoms were provided to all newly diagnosed persons and all other baseline services are kept (Table 2). In the other set of scenarios beside the previous scenario of 80% testing coverage among youth with TasP/condoms, gender-age-tailored interventions for susceptible youth are combined at various coverage and/or efficacy values (Table 3).

In the following sections, we studied the role of youth in the Nyanza HIV epidemic. We then compared two sets of youth-focused HIV strategies to adult-focused HIV strategies with 40%, 50%, 60%, 70%, and 80% adult HIV testing coverage maintained over the years with condoms and TasP administered for HIV-diagnosed adults. The first set of youth-focused HIV strategies included focusing further testing on youth and providing TasP to HIV-diagnosed youth and condoms to all HIV-diagnosed persons (TasP/condoms) (Fig 2 and Table 2). The second set of comparison scenarios included a more comprehensive package encompassing 80% youth-testing coverage with TasP/condoms and with and without other interventions (MC, PrEP, and CT) for HIV-susceptible youth comprising 60 alternative combinations of gender-tailored prevention interventions (Fig 2 and Table 3).

We recorded the following three population level outcomes for each scenario to facilitate comparison: cumulative new HIV infections, cumulative DALYs and the cumulative costs required to achieve the desired coverage levels.

### Sensitivity analyses

Table 1 lists our model assumptions in terms of default parameter values, which are fitted, reported in the literature, or empirically measured; to span a plausible range for these parameters we used two other values for each. We performed a one-way sensitivity analysis to assess the robustness of predicted difference between two intervention strategies in terms of the difference in the impact on DALYs averted, HIV infections averted, and costs. We examined the sensitivity to variations of 1) infectivity on treatment, 2) survival years on untreated HIV, 3) dropout rate from HIV treatment for youth, 4) excess mortality on treatment compared to uninfected persons with the same age, 5) assortativity in sexual risk mixing, 6) effective sexual partner change rate, 7) assortativity in age mixing, 8) maximum age difference between partners.

## Results

We estimated the role of youth in the HIV epidemic in Nyanza by calculating the proportion of attributable sexual transmissions from and to youth between 2004–2014. While 15-24-year-old youth represent about 17% of the population in Nyanza, the fraction of total incident infections, in the model, attributable to young index partners (aged 15–24 years) is about 26% from females and 4% from males. Between the years 2004–2014, an estimated 41% of new infections from sexual transmission among adults in Nyanza were among 15–24 year old females and 9% among 15–24 year old males. The 5% estimated cumulative risk of HIV for girls who were 15 years old in 2014 will more than triple to 18% upon reaching age 25 and will increase 9-fold (44%) by the end of life. We estimated a mean age at infection of 24 for females and 27 for males.

### The potential of focusing treatment as prevention on youth

**Fig 3:**
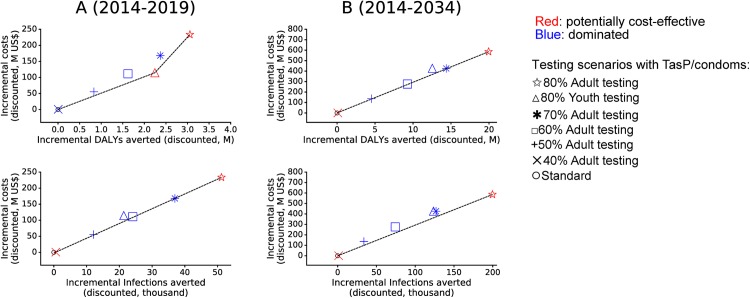
**Comparison of cumulative impact and costs over (A) 5 years and (B) 20 years of youth-focused scenario (upward triangle) to adult-focused scenarios.** The youth-focused scenario consists of 80% testing coverage among youth and providing TasP and condoms to HIV-diagnosed youth as well as prompt treatment at CD4≤350 cells/mm^3^ and condoms for HIV-diagnosed adults. Adult-focused scenarios encompass increased testing among adults and providing only TasP and condoms. The details of the six intervention scenarios compared here are in Table 2. The resulting cost frontiers (lines through red points) from cost-effectiveness analyses using DALYs averted (top-row) and infections averted (bottom-row) are shown along with the dominated scenarios (blue points). The estimates are shown at year 5 in panel A and at year 20 in panel B. Dominated scenarios comparatively cost more US$ per DALY averted (top-row) or more US$ per infection averted (middle-row). M = million.

Our analyses in the top row of Fig 3 compare focusing high HIV testing coverage with TasP and condoms on youth to focusing on adults in Nyanza. Assuming equal program costs regardless of youth vs. adult focus, short term predictions (over 5 years) indicate that focusing on youth would be the cheapest cost-effective scenario to avert DALYs. The youth-focused scenario performed better in averting new HIV infections than adult scenarios with testing coverage up to 50%. In the longer term (over 20 years), the youth-focused scenario performed better than the adult scenarios with testing coverage up to 60%, and approached the performance of adult scenarios with 70% coverage.

### The potential of a comprehensive prevention intervention package focusing on youth including HIV-uninfected persons

**Fig 4:**
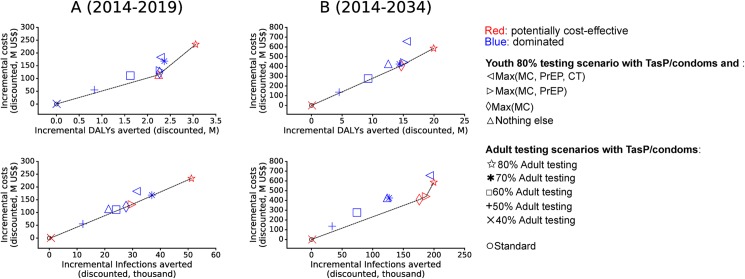
**Comparison of cumulative impact and costs over (A) 5 years and (B) 20 years of youth-focused to adult-focused scenarios.** Youth-focused scenarios are based on 80% testing coverage among youth and providing TasP for HIV-diagnosed youth and condoms to newly diagnosed persons in addition to age-gender-risk targeted interventions to HIV-susceptible youth. Adult-focused scenarios encompass increased testing among adults and providing only TasP and condoms following diagnosis with HIV. The comparison involved 65 scenarios but in the plots, only potentially cost-effective scenarios (red colored points) and selected dominated scenarios (blue colored points) are shown. Cost frontiers (lines through red points) are shown in terms of DALYs averted and infections averted (top and bottom figures, respectively) at 5 and 20 years (A and B panels, respectively). M = million.

Here we carried the comparison with the adult-focused scenarios when gender-tailored prevention interventions for HIV-susceptible youth (described in Table 3) are added to the base youth scenario of youth testing at 80% coverage and providing TasP and condoms for HIV-diagnosed youth. We compared the costs and the impact on DALYs and new infections of these interventions by comparing 65 scenarios (selected scenarios are shown in Fig 4).

In the short term (over 5 years), a comprehensive youth package with optimized MC and PrEP performed better than adult scenarios with adult testing up to 60%, both in averting DALYs and new HIV infections. In the long term (over 20 years), the comprehensive package having high testing coverage among youth with TasP/condoms and optimized uptake of MC among HIV-susceptible young men was the cheapest potentially cost-effective package. This package outperformed the adult scenarios with TasP/condoms up to 70% adult testing. The addition of high uptake of PrEP among high risk 20–24 year-old women averted 0.5 million more DALYs and 11,000 more new infections and cost 31.8 million more US$ over 20 years. This suggests that if optimizing PrEP coverage and adherence among HIV-susceptible high-risk young girls is possible at the assumed cost of $200 per person per year and in combination with youth-focused TasP/condoms and MC then such usage of PrEP would be cost-effective in reducing HIV incidence compared to adult TasP/condoms scenarios with testing coverage up to 70% (Fig 4).

**Fig 5:**
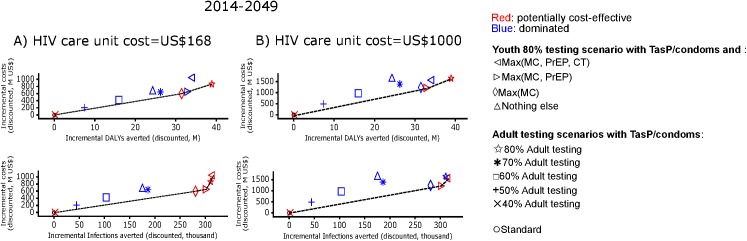
**Comparison of cumulative impact and costs over 35 years of youth-focused to adult-focused scenarios with (A) default annual care costs per person in late stages of HIV and (B) when these care costs are much higher.** The comparison involved 65 scenarios but in the plots, only potentially cost-effective scenarios (red colored points) and selected dominated scenarios (blue colored points) are shown. Cost frontiers (lines through red points) are shown in terms of DALYs averted and infections averted (top and bottom figures, respectively) at 5 and 20 years (A and B panels, respectively). M = million.

Over the years, youth-focused TasP/condoms with optimized MC or optimized MC and PrEP would be cost-saving in averting HIV infections and in averting DALYs compared to adult-focused TasP/condoms scenarios (Fig 5A). Over the years and with higher HIV care cost than we assumed (US$1000 instead of US$168 in late stages of HIV), youth-focused HIV testing with TasP/condoms and optimized MC and PrEP would be the most efficient intervention in averting HIV infections and DALYs that would be cost-saving even when compared to adult-focused TasP/condoms scenarios with testing coverage up to 80% (Fig 5B). Also, at higher HIV care costs and over longer terms, a comprehensive package focusing on youth including optimized uptakes of MC, PrEP and the gender-age-school-tailored CT (with 90% coverage and reduction in partnerships with older men) among youth would be more cost-effective in reducing HIV incidence than the scenario with 80% adult testing (Fig 5B).

These results indicate that at least over the next 35 years–when today’s young persons in Nyanza reach the age of 50 –youth-focused HIV testing with HIV treatment at diagnosis for youth and at least at CD4≤350 cells/mm^3^ for adults, combined with condoms for newly-diagnosed persons and circumcision focusing on HIV-negative young men following HIV testing, might be the optimal cheapest package that averts the most HIV infections and best reduces HIV risk (by reducing HIV incidence among youth and in the population, Figure E in S1 File) while cost-effectively averting substantial DALYs over the years.

### Sensitivity analyses

We performed one-way sensitivity analyses (Figure G in S1 File) to assess the robustness of our predictions on the impact and costs of youth-focused 80% HIV testing with TasP/condoms and optimized MC for HIV-negative young men as compared to an adult-focused 70% HIV testing with TasP/condoms over 5 and 20 years. Our choice for the adult-focused intervention was informed by our results.

Our prediction in Fig 4 that the youth-focused intervention compared to the adult-focused intervention saves money but averts fewer DALYs and fewer infections over the short term of 5 years were mostly robust against the assumed variations in model parameters. Over 20 years, our prediction that the youth-focused intervention averts more new infections than the adult-focused intervention is robust against the variation in model parameters; however, the excess number of DALYs it generates over 20 years were sensitive to the variation in the maximum age difference between partners, the effective partner change rate, the assortativity in sexual-risk mixing and the excess mortality on ART.

## Discussion

We followed a modeling approach that compared youth-focused HIV-prevention and epidemic control interventions to adult-focused interventions based on increased HIV testing. Our results indicate that focusing high coverage HIV testing on youth, providing TasP to HIV-diagnosed youth and condoms to newly diagnosed persons and 80% MC uptake among uncircumcised HIV-susceptible young men, as well as continued ART at CD4≤350 to HIV-diagnosed adults, is more efficient in averting new HIV infections and in decreasing DALY burden over the long term (≥35 years) compared to adult-focused scenarios of HIV testing with up to 70% coverage with condoms and TasP following HIV diagnosis. Our findings reflect that the potential cost-effective role of youth-focused interventions increases especially over the long-term in those situations when it is not feasible to provide low-cost programs encompassing high coverage HIV testing exceeding 70%, HIV care, TasP and condoms to HIV-infected adults. Particularly, when HIV care costs are high, additional spending now on averting new HIV infections by focusing HIV prevention on youth becomes more important because it pays off over the years by decreasing the number of persons in need of care.

Because of the large uncertainty in TasP program costs, we assumed equal program costs for TasP regardless of the testing focus or the level of coverage. This allowed us to exclude these costs in the comparison. If TasP program costs, contrary to our assumption were more expensive when high testing coverage was focused on adults compared to youth focused testing, then our results would still be valid. However, if the costs of managing a youth-focused TasP program with high uptake of testing surpasses the costs of adult-focused programs with high testing coverage, then our results might not be valid.

In this modeling exercise our focus was not to assess the implications of lower ART adherence levels such as regimen failure and the need to use costly second line therapy as well as the risk of developing and transmitting HIV resistant strains; thus we assumed theoretical conditions where persons who are on HIV treatment have either perfect adherence or leave treatment and thus, zero adherence. In the real world context, adherence measurement [66] and supportive counseling addressing barriers to adherence [66,67] would be key to achieve high adherence and viral suppression levels among persons on HIV treatment. In our estimates of PrEP impact, the high efficacy (= 90% reduction in HIV acquisition) and concomitant high adherence among high risk 20–24 year-old women were important for a potential cost-effectiveness role of PrEP. If adherence/efficacy were lower than anticipated among this high risk age-cohort of girls, then our estimates of the impact of PrEP would be lower. We assumed that persons who are diagnosed with HIV are linked to HIV care and to risk reduction counseling (increased condom usage) and to HIV treatment according to the treatment scenario under consideration. This might be a strong assumption considering suboptimal linkage to care in many settings in sub-Saharan Africa. However, we considered HIV care in order to factor in its costs because these costs proved to be important in our analyses to differentiate the cost-effectiveness of different scenarios. One of the limitations in our study is the optimistic assumption that 96% reduction in onward transmission is experienced by persons on HIV treatment. Recent results show that only virally suppressed patients on ART would have such reduction [68–71].

We compared our model estimates against previously published models in the literature with a caveat that most modeling work has not compared youth-targeting interventions to adult interventions. A comparison with incidence predictions using Nyanza sexual behavior data and the Spectrum modeling package [72] reveals a comparable incidence among adults in Nyanza (1.3% versus 1.1% per person year for fitted case in our model).

We compared our predicted cost-effectiveness ratios (ICERs) to estimates of ICERs comparing treatment initiation criteria over 5 years in Walensky et al. [73] using similar costs and found our ICERs are about one order of magnitude lower. The difference may be attributed to (1) our possible overestimation of average lost life years in calculating DALYs for some age groups by setting it equal to the inverse value of the per capita mortality rates (however, scaling these values by a common factor (<1) increased the value of the ICERs but did not affect the order in which the scenarios appear, data not shown), (2) differences in the epidemiological context, or (3) the fact that in Walensky et al. there are assumptions including no impact of treatment on prevention. By comparing our model estimates of adult-focused TasP and ART at CD4≤≤350 in context of high adult HIV testing coverage using similar costs for HIV care as in the Modelling Consortium study for Zambia [40] we found TasP dominates ART (i.e., the ICER reveals cost-saving with larger DALYs averted) just as was found by the Consortium study. However, in our study we assumed HIV care costs for Kenya (following [41]) that were much lower than Zambia's costs in the Consortium study.

Our findings for PrEP’s role in comparison with TasP/condoms and MC are in-line with earlier findings considering TasP and MC interventions for the wider adult population that showed that offering PrEP (albeit without high-risk targeting in that study) to young women and men would have less priority but would avert extra HIV infections if implemented in combination with other prevention interventions [74]. We found that the incremental benefit of PrEP in combination prevention with TasP, condoms and MC would be always cost-effective in reducing HIV incidence compared to adult scenarios with testing coverage up to 70% and its additional impacts on both HIV incidence and DALYs become more cost-saving over the years when the care costs are higher. It is worth noting that Kenya is now one of the first sub-Saharan African countries to roll out PrEP (a Gates-funded project will make PrEP available to n = 20,000 Kenyans in 2017).

### Policy implications

Access by youth to HIV prevention, testing, and ART has been recognized to be a key constraint in the goal of HIV reduction and ‘ending the epidemic [75]. Given that the HIV profile of youth in Nyanza might be similar to their HIV profiles in other high-HIV-burden generalized epidemic African settings, our results have implications for HIV prevention programs in these settings. Our model shows that focusing on youth would be cheaper and have larger impact on averting HIV new infections and HIV mortalities over time than widespread adult interventions unless very high adult testing coverage with immediate treatment occurs. The best strategies are those that can substantially reduce both HIV incidence and HIV mortality reducing the scale of the HIV epidemic while being a less expensive alternative. We showed in this modeling work that an optimal approach in a high-HIV epicenter like Nyanza is to continue offering condoms and ART at least at CD4≤350 following HIV diagnosis at current testing rates and to focus further HIV testing coverage on youth, optimize medical circumcision among HIV-negative young men and coverage and adherence of PrEP among high risk young women and offer immediate linkage to care and treatment with risk reduction counseling for newly diagnosed persons with HIV. On a wider scale, these results show that it might be cost-efficient to invest strategically in prevention interventions focusing on young people.

Further research on age targeting compared to other strategies would be necessary to determine the optimum age-targeting strategy for a given HIV-epidemic setting. In an era where resources for HIV programming are plateauing, with ambitious targets for epidemic control, consideration of age targeting may be useful.

## Supporting information

S1 File A file containing model equations and more details on parametrization and calibration of the model and additional results. (DOCX)

## Abbreviations

TasP (treatment as prevention): Antiretroviral therapy (ART) treatment initiation at HIV diagnosis, supporting primary transmission risk reduction and secondary/tertiary prevention of HIV disease progression
US$: United States of America Dollars in 2012
