# Supplementary material for: The potential impact and cost of focusing HIV prevention on young women and men: A modeling analysis in western Kenya
Source: PLoS One. 2017 Apr 12;12(4):e0175447. doi: 10.1371/journal.pone.0175447 (PMC5389814; doi:10.1371/journal.pone.0175447)
Supplement: S1 File — (DOCX) [file pone.0175447.s001.docx]

## Supplementary Information Appendix

## **The potential impact and cost of focusing HIV prevention on young women and men: a modeling analysis in western Kenya**

Ramzi A. Alsallaq (RAA), Jasmine Buttolph (JB), Charles M. Cleland (CMC), Timothy Hallett (TH), Irene Inwani (II), Kawango Agot (KA), Ann E. Kurth (AEK)

In this document, we provide further details to the main text, arranged in the following sections:

1. **Model equations**
2. **Model parameterization and calibration**
3. **Cost assumptions**
4. **Additional results**

# A. Model Equations

The model is deterministic and compartmental and consists of a system of differential equations that were implemented and solved in Matlab (Natick, Massachusetts, USA). All used software programs are available by request from the corresponding author.

The equations describe the change in the number of susceptibles ($X$), HIV infecteds ($Y$), and individuals with AIDS ($A$) of both genders (men and women) with respect to time $t$. In addition to gender/sex ($k$=1 for uncircumcised males, $k$=1 for circumcised males, $k$=3 for females not on cash transfer, $k$=4 for females on cash transfer), the model is stratified to three sexual risk activity groups ($l$=1 (highest) to 3 (lowest)), fourteen five-year age groups ($i$=1 (<5) to $i$=14 (≥69)), HIV-testing status ($n$=1 (never tested), $n$=2 (tested and increased condom use), $n$=3 (tested and no increase in condom use)), PrEP status ($p$=1 for persons not on PrEP, $p$=2 for persons on PrEP), and eligibility for female-specific interventions ($o$=1 (eligible), $o$=2 (not eligible)). It has the following structure:

-----(1)

Here $\mu_{ki}$is the gender and age specific per capita death rate; $\lambda_{klipno}(t)$ is the force of infection per individual of that gender, age, risk activity group, PrEP status, test status, and eligibility status for female-specific interventions and at time $t$; the quantities ($\omega_{si}^{-1}$,$s$=1,..5) represent the average number of years spent in the infected stages $Y_{klipno}^{s}$ by an individual of age $i$; the parameter$\alpha$represent the per capita growth rate before introducing the HIV; the distribution of the population in each category is given by $\Omega_{klipno}$; while $\Delta a$represent the size of each age group in years. The quantity $\delta_{ij}$ is only non-zeros and equal 1 when $i=j$, and the quantity $K_{ij}$is only non-zero and equal 1 if $j\geq i$.

The five infected stages represent the variable infectiousness and the declining CD4 count as infected individuals progress through the incubation period. The first stage $Y_{klipno}^{1}$is the acute stage with onward transmission per sexual act heightened by a factor$f_{\beta}^{1}$ with respect to stage 4; $Y_{klipno}^{2}$represents the stage in which CD4 >=350 and having the same infectiousness as in stage 4; $Y_{klipno}^{3}$ represents when CD4 between 250 and 350 and the same infectiousness as in stage 4; $Y_{klipno}^{4}$ represents CD4 between 100 and 250 and onward infectiousness per sexual act of $\beta_{0}$; and $Y_{klipno}^{5}$represents CD4 below 100 and before full blown AIDS with onward transmission probability per sex act that is $f_{\beta}^{5}$ times that in stage 4.

Individuals initiating HIV treatment upon HIV-diagnosis move to $\bar{Y}_{klipno}^{1}$while those initiating treatment at CD4=350 cells/mm^3^ and below move to $\bar{Y}_{klipno}^{2}$and $\bar{Y}_{klipno}^{3}$; respectively. Persons initiating HIV treatment at CD4>350 cells/mm^3^ or at CD4<350 cells/mm^3^ can drop out and go to $\bar{Y}_{klipno}^{4}$and $\bar{Y}_{klipno}^{5}$; respectively. We discuss the parameters in the equations containing these state variables below when discussing treatment interventions.

The initial numbers in each category ($\Omega_{klipno}\times N_{0}$) are determined by distributing the initial size of the population $N_{0}$ over the categories using the following quantities: the sex ratio of individuals at birth $\nu_{k}$, the proportion of persons in this sexual activity $\eta_{l}$, the proportion of persons of this gender in this age group $\psi_{ki}$, the proportion of men that are circumcised at childhood or adolescence $\pi_{k}$, the proportion of females who are eligible for female-devoted interventions (PrEP and cash transfer or CT) $\iota_{kpo}$, and the initial number of the infected individuals (the seed) which we assumed to be a small number of uncircumcised men of risk category 3, age between 25 and 29, and in infection stage 2. Initially $\iota_{f}$ of females are eligible for female-specific interventions respectively ($\iota_{kpo}$=$\iota_{f}$ for $k$=3, $p$=1, $o$=1; $\iota_{kpo}$=1-$\iota_{f}$ for $k$=3, $p$=1, $o$= 2) and females initially are in no PrEP and no CT categories ($\iota_{kpo}$=0 for $k$=4 or $p$ =2 irrespective to the value of$o$) while the population is in category 1 for testing ($n$=1).

At each moment of time, a number of newborns are introduced in each gender, sexual activity group, and eligibility group to female-specific interventions:

--(2)

Here $i$=1 indicates zero age; $a_{1}$ is the age of commencement of sexual activity; $a_{2}$is the age of cessation of sexual activity; $k$=3,4 implies women; $\nu_{k}$is the sex ratio of individuals at birth; $\eta_{l}$is the proportion of infants born into this sexual activity; $\xi$is the mother-to-child transmission probability; $\pi_{k}$ (=0; $k$=3,4 ) is the proportion of babies circumcised before growing to sexual debut; $\iota_{kpo}$is the proportion of babies that will grow up to be eligible for female-specific (PrEP and CT) interventions; and $\varphi_{a}$is the per capita yearly fertility rate of women in age group $a$.

The per capita force of infection $\lambda_{klipno}$ is defined for the sexually active population ($i$≥3) by

-------(3)

Here is the reduction in the risk of HIV acquisition because of circumcision (non-zero for $k$=2); is the reduction in the risk of HIV acquisition because of PrEP (non-zero for $p$=2); $C_{kli}(t)$ is the total number of partnerships formed per year by individual of gender $k$ risk activity $l$ age $i$. Time dependency of $C_{kli}(t)$ is used to reflect the reported reduction in sexual partners in Kenya between 1998 and 2003 [[1](#_ENREF_1),[2](#_ENREF_2),[3](#_ENREF_3)]. The term $\rho_{kll'ij}$ is the probability that someone of gender $k$risk activity $l$ age $i$ will have a sexual partner of the opposite sex in the activity group $l'$of age $j$. Persons with AIDS are not considered sexually active; thus excluded. The terms $z_{sk'l'ljin'np'po'o}$ and $\bar{z}_{qk'l'ljip'pn'no'o}$ give the transmission probability from an infected person of gender $k'$ sexual activity $l'$ age $j$ PrEP status $p'$ test status $n'$eligibility to female-specific interventions $o'$ in HIV stage $s$ or treatment stage $q$ per partnership with a susceptible person of the opposite gender in sexual activity $l$ age $i$ PrEP status $p$ test status $n$and eligibility to female-specific interventions $o$ and is defined including the effect of condom use as follows:

------------(4)

Here $\beta_{0}$ is the per sexual act probability of transmission from a person in the chronic phase of the infection at stage 4; $f_{\beta}^{s}$, $\bar{f}_{\beta}^{q}$, $f_{\beta}^{k}$, and $f_{\beta C}$are the factors change from the probability of transmission in chronic phase from a person in the HIV stage $s$, in treatment stage $q$, gender $k$ and when the sexual act is condom protected, respectively; $\Pi_{ll'}$ is the total number of unprotected sexual acts in a partnership between individuals of these sexual activity groups; and $P_{cll'ijnn'}$ is the probability that condom is used correctly in a sexual act between a susceptible person in the activity group $l$ age $i$ test status $n$ and an infected partner of activity $l'$ age $j$ and testing status $n'$.

In what follows the definitions of $C_{kli}(t)$ and $\rho_{kll'ij}$are presented assuming no CT intervention (no females in category $k$=4) but we introduce expressions without losing generalizability. We derive $C_{kli}(t)$ from the mean partnership acquisition rate $M_{ki}(t)$ of the individual of gender $k$ age $i$ by requiring:

$M_{ki}=\sum_{l=1}^{3} \eta_{l}C_{kli}$------------------------(5)

This can be inverted, by requiring that the rates of various activity groups are just the multiplicative of the rate of the first activity group i.e. $C_{kli}=\tau_{l}C_{k1i}$ and $\tau_{1}=1$, to:

$$C_{k1i}=\frac{M_{ki}}{\eta_{1}+\eta_{2}\tau_{2}+\eta_{3}\tau_{3}}$$

$C_{k2i}=\tau_{2}C_{k1i}$------------------------(6)

$$C_{k3i}=\tau_{3}C_{k3i}$$

The probabilities $\rho_{kll'ij}$ are required to satisfy $\sum_{j=a_{1}}^{a_{2}} \sum_{l'=1}^{3} \rho_{kll'ij}=1$, for sexually active persons of gender $k$ sexual activity $l$ and age $i$, and can be expressed as the multiplication of two parts as follows:

---------(7)

Here $\varepsilon_{r}$ is the tendency of an individual to form sexual partnerships with partners from the same sexual activity group (assortativity in sexual-risk mixing); $\varepsilon_{ki}$is the tendency of a person of gender $k$ and age $i$ to form sexual partnerships with partners who are the same age (assortativity in age mixing); $F_{k}(i)$ represent the set of opposite sex age groups that someone of gender $k$ age $i$ can sexually mix with; $R_{kll'i}^{r}$and $R_{klij}^{a}$are, respectively, the risk- and age- proportionate components of mixing for someone of gender $k$ sexual activity $l$ age $i$ with partners of sexual activity $l'$ and age $j$. These proportionate components are given by:

-----------(8a)

-----------(8b)

Here $S_{k'\to kli}$ represents the total supply of partnerships per year from persons of gender $k'$to opposite sex partners of risk activity $l$ age $i$. Males choose younger females partners up to 10 years younger, while females choose older males up to 10 years older. Mixing of young men with older women is ignored because while it occurs, it is not the common norm in Kenya [[4](#_ENREF_4),[5](#_ENREF_5)].

We apply balancing of partnerships between males and females by calculating the ratio of partnerships:

-------------(9a)

and requiring that:

$\begin{matrix} \rho_{1ll'ij}\to\rho_{1ll'ij}\times D^{\theta-1} \\ \rho_{3l'lji}\to\rho_{3l'lji}\times D^{\theta} \end{matrix}$ ----------------(9b)

Here $\theta$ ($0\leq\theta\leq1$) is a parameter that determines which gender is controlling the balance, such that a value of 0.5 ensures a midpoint balance.

# B. Model Parameterization and Calibration

### Model Calibration

In calibrating the fitted case we varied the values of the most uncertain parameters related to sexual behavior within plausible ranges as in Table E and we compared model prevalence to data. Out of 75,000 different parameter sets that were tried, only 13 fit age-gender stratified prevalence data. We chose one of the parameter sets at random to represent our model fit (Fig 1B in main text). It is to be noted that keeping age-specific survival time on infection before death fixed as in Todd *et al* [[6](#_ENREF_6)] and comparing prevalence trends obtained based on reported behavioral data from Nyanza, model prevalence cannot reproduce data prevalence points without assuming reduction in sexual behavior in all sexual activity groups of the population. Reports from Kenya at large indicate reduction in partner acquisition rates among young males and females in urban and rural communities during the period 1998-2003 [[1](#_ENREF_1)]. During the same period there was more than 60% reduction in the percentage of men and women who had two or more sexual partners in the 12 months before the surveys KDHS 1998 and KDHS 2003 [[2](#_ENREF_2)] (The NASCOP report in 2005 compared sexual risk between KDHS 1993, 1998, and 2003 and reported significant decline in the proportion of men and women who have multiple sexual partners).

Table A: Model fitting parameters and their ranges

| Parameter | Range | Fitted value | Resource |
| --- | --- | --- | --- |
| Assortativity in sexual risk mixing | 0.2-0.9 | 0.89 | Representative range |
| Assortativity in age mixing | 0.1-0.4 | 0.3 | [[7](#_ENREF_7)] |
| Year reduction in partners started in Kenya | 1998-2000 | 2000 | [[8](#_ENREF_8)] |
| Percentage reduction in partners | 0-50% | 48% | [[2](#_ENREF_2),[8](#_ENREF_8)] |
| Number of years over which reduction in partners took place | 3-5 | 4.5 | [[8](#_ENREF_8)] |
| Fraction of the population in the low risk activity group | 0.6-0.99 | 0.75 | Representative |
| Fraction of the population not in the low risk activity that are in the high risk activity group | 0.01-0.9 | 0.045 | Representative |
| Relative partner acquisition rate (high to low) | 40-125 | 116.7 | Representative |
| Relative partner acquisition rate (intermediate to low) | 5-25 | 8.8 | Representative |
| Proportion of sex acts adequately protected by condoms in partnerships involving no high risk individuals | 0-0.1 | 0.0008 | Representative for marital partners [[9](#_ENREF_9)] |
| Proportion of sex acts adequately protected by condoms in partnerships with low or intermediate risk individuals involving a high risk individual | 0.1-0.3 | 0.16 | Representative for non-marital partners [[9](#_ENREF_9)] |
| Proportion of sex acts adequately protected by condoms in partnerships with high risk individuals involving a high risk individual | 0.3-0.8 | 0.52 | Representative for non-regular partners [[9](#_ENREF_9)] |
| Mean partner acquisition rate for 20-24 men | 3-7 | 4.0 | among all risk categories |
| Mean partner acquisition rate for 20-24 women | 3-5 | 3.6 | among all risk categories |
| Year HIV epidemic started in Nyanza | 1982-1985 | 1984.5 | [[10](#_ENREF_10)] |

###
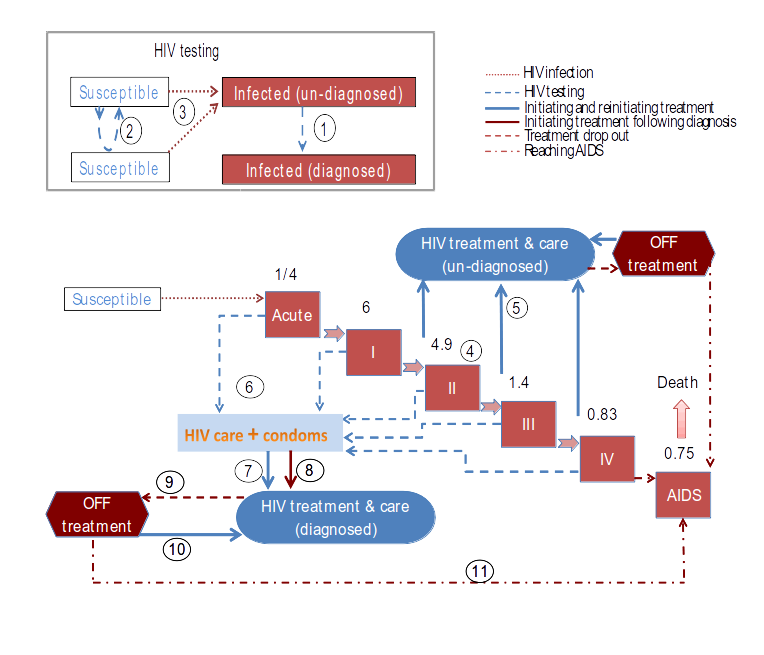


### Figure A: The HIV dynamical transmission model representative of the HIV epidemic in Nyanza and its calibration. Implementation of HIV testing (inset) and implementation of HIV pathogenesis, HIV care and treatment and condom intervention. Individuals who have never been diagnosed with HIV are subject to a random annual testing rate. HIV infected persons get diagnosed (1) and no longer tested whereas susceptibles can be tested again (2). Susceptibles upon infection (3) belong to the undiagnosed infection category. Undiagnosed infected persons when not treated go into the acute HIV stage first and then progress through five other stages including AIDS (4) spending mean intervals in each stage that vary generally by age at infection (shown in the figure for a 15-24 year-old person). In the fitted scenario, because HIV care and treatment are not based on HIV diagnoses, an estimated 10% of those with CD4≤350 cells/mm^3^ initiate HIV treatment at CD4=350 cells/mm^3^ and the rest at lower CD4 count (5). In standard and comparison scenarios HIV-diagnosed persons link to care and decrease condom non-use with sexual partners who are susceptible or with unknown status (6) and initiate ART (promptly following diagnosis at CD4≤350 cells/mm^3^) (7). In the scenarios focusing TasP on youth, HIV-diagnosed youth initiate treatment immediately following diagnoses (8) in addition to the standard ART for older diagnosed persons (7). In-care and on-treatment persons stay so until death or until they drop out of treatment (9), and those in care but not on treatment progress through disease stages until they become eligible and then initiate treatment. Re-initiating treatment (10) is possible for dropouts with CD4≥250 cells/mm^3^ otherwise they progress to AIDS (11). Model parameters are calibrated using epidemiological and behavioral data from Nyanza.

### Model representation of interventions and calibration of historical levels

### 1. Testing and Condoms

As mentioned after Eq.1 HIV testing is implemented by stratifying the population into three categories by the index $n$ ($n$=1 (never tested), $n$=2 (tested and increased condom use), $n$=3 (tested and no increase in condom use)). Starting from a time $t_{0}$, we adjust the HIV testing rate (as likelihood per capita per year) using a parameter $\zeta_{1}$ and from a later time $T_{0}\geq t_{0}$we adjust HIV testing rates depending on the treatment intervention which is either among youth (as likelihood per young individual per year) or among adults (as likelihood per adult per year) using another parameter $\zeta_{2}$ ($\zeta_{2}$≥$\zeta_{1}$). These two parameters control the numbers moving per unit time to category 2 (testing rates).

$\zeta_{i}(t)=\left\{\begin{matrix} \zeta_{1};\text{ }t\geq t_{0},\text{ }i=1,...,14 \\ \zeta_{2};\text{ }t\geq T_{0}>t_{0},\text{ }i=4,5\text{ or }i\geq4 \end{matrix}\right.$-----------------------(10)

Where $T_{0}$ represents the time at which interventions start. Thus for $t\geq t_{0}$ we have HIV testing for the population (susceptible $X_{klipno}$and infected ) given by the equations:

------------------------(11a)

-------------------------(11b)

Where for the susceptibles no subsequent differences between category 1 and 3, while infecteds who are newly diagnosed with HIV move to category 2 and are removed from the pool of population who are exposed to testing for HIV status in the future (Figure A (inset)).

Infecteds in testing category 2 receive counseling for reducing HIV risk by reducing condom non-use to protect their partners who are uninfected or with unknown HIV status. When recruited for risk reduction intervention, they sustain this change in behavior for a mean duration that depends on age (set infinitesimally small for non-targeted age groups to make no difference between testing categories 2 and 3). This intervention is implemented by a parameter $œ$ (representing the relative reduction in condom non-use for sexually active population in testing category 2 with respect to baseline condom non-use established in the fitted case) and updating the probability $P_{cll'ijnn'}$ in Eq.4 that condom is used correctly in a sexual act between a susceptible person in the activity group $l$age $i$ test status $n$ and an infected partner of activity $l'$ age $j$ and testing status $n'$ by

$\begin{matrix} P_{cll'ijnn'}\to1-(1-œ_{n'}(t))(1-P_{cll'ijnn'}) \\ œ_{n'}(t)=\left\{\begin{matrix} 0;\text{ }t<T_{0}\text{ or }n'\neq2 \\ œ;\text{ }t\geq T_{0}\text{ and }n'=2\text{ } \end{matrix}\right. \end{matrix}$ ----------------------------(12)

### 2. Treatment

Treatment initiation parameters in Eq.1 ($\varsigma_{kin}^{q}$;$q=1,...,4$) control proportion of individuals of sex $k$ age $i$ testing categories $n$ starting treatment and moving to treatment compartments $\bar{Y}_{klipno}^{1}$, $\bar{Y}_{klipno}^{2}$, and $\bar{Y}_{klipno}^{3}$; $ß_{l}$ and $ß_{e}$specify excessive mortality of persons starting treatment late (CD4<350 cells/mm^3^) and early (CD4≥350 cells/mm^3^ ) respectively; $\vartheta$specify dropout rate from treatment; $\bar{\omega}_{d}$specify duration spent in drop out compartments $\bar{Y}_{klipno}^{4}$and $\bar{Y}_{klipno}^{5}$.

The parameters ($\varsigma_{kin}^{q}$;$q=2,3,4$) give three opportunities to promptly initiate treatment by any person of sex $k$ age $i$ testing categories $n=1,2,3$ and his/her CD4≤ 350 cells/mm^3^. These are: whenever his/her CD4 becomes exactly equal to 350 cells/mm^3^ ($q=2$), after age-dependent mean times spent in stage II ($q=3$), or after age-dependent mean times spent in stage II and III from the time his/her CD4 reaches 350 cells/mm^3^ ($q=4$). The last two instances correspond to CD4=250 and CD4=100 cells/mm^3^, respectively (Figure A).

Before 2014, treatment in the model is scaled up starting at 2002 (the year HIV treatment became available in Nyanza [[11](#_ENREF_11)]) for those with CD4≤350 cells/mm^3^. To calibrate model parameters ($\varsigma_{kin}^{q}$;$q=2,3,4$) using reported numbers initiating ART at CD4≤350 cells/mm^3^, we assume (1) they are equal to reflect equal likelihood of initiating ART at any of the three chances (2) with adult women having twice the likelihood of initiating ART at CD4=350 compared to same age men and (3) no coupling between HIV testing and treatment because current HIV treatment practice in Kenya is not based on CD4 screening. By iteratively comparing the cumulative number of persons initiating ART between 2002 and 2009[[12](#_ENREF_12)] and in year 2011[[13](#_ENREF_13)] with model produced numbers as illustrated in Fig 2B and 2C in main text, model parameter values are calibrated. To translate the age/sex stratified numbers initiating ART during 2011[[13](#_ENREF_13)] according to WHO stages I, II, III, and IV to corresponding numbers initiating based on CD4≤350 cells/mm^3^, we utilized likelihoods of having CD4≤350 cells/mm^3^ given WHO stage in similar settings [[14](#_ENREF_14)].

Treatment initiation in intervention scenarios is represented based on knowledge of HIV status; therefore, those in categories $n$=2 and $n$=3 categories can initiate treatment once they meet treatment criteria. In the main text, we consider two criteria for treatment following HIV diagnoses: (1) At CD4≤350 cells/mm^3^ and (2) Immediately upon HIV-diagnosis. For the first criterion, the parameters ($\varsigma_{kin}^{q}$;$q=2,3,4$) are set at 100% for the targeted age and in addition to this for the second criterion, the parameter $\varsigma_{kin}^{1}$is set large enough for the targeted age (e.g. newly diagnosed youth with HIV) to represent immediate initiation of HIV treatment/ART and zero for other age groups. Otherwise, treatment without diagnosis can be initiated mostly late at CD4≤250 as established by fitting the model to treatment initiation data from Nyanza in the fitted case (see model fitting and calibration).

Persons who are HIV-diagnosed (testing categories $n=2,3$) and have not initiated treatment are assumed to be in HIV care with appropriate CD4 monitoring.

### 3. Male circumcision (MC)

We incorporated the influence of male circumcision (MC) by dividing the male population into circumcised and uncircumcised. Circumcised men experience a drop in the hazards of acquiring HIV infection with respect to time periods when uncircumcised, as quantified by the quantityas indicated in Eq. 3.

Up to year 2009 MC prevalence in Nyanza was 48% [[15](#_ENREF_15)] and we represented it as occuring at adolescence; we used this proportion to define $\pi_{k}$for $k$=2 in Eq.2 which otherwise is zero. To calibrate the model to recent progress in scaling up MC (after year 2009) we incorporate the MC intervention in the fitted case among males 15-24 (irrespective to HIV status as data show) because most of the recent reported progress is among this age category [[2](#_ENREF_2)]. We assumed that the rate of MC uptake in the fitted case continues in future years (as in Fig 1D in main text) unless there is an intervention to enhance uptake as specifically assumed among newly tested HIV-negative young men in the main text.

Thus, further MC among young men in the intervention scenarios is generated by moving newly tested HIV-uninfected young men (testing category $n$=2) from category $k$=1 to category $k$=2. The following equations describe MC levels in before MC intervention (time<$T_{0}$) and after the MC intervention (time≥$T_{0}$):

------------------------(13a)

-----------------(13b)

-----------------(13c)

Here $ç_{b}$represents the movement from the uncircumcised to the circumcised categories when MC is not based on testing as in the fitted case while $ç_{v}$is the parameter that controls the movement in the intervention scenarios when MC is based on new HIV testing. The time $t'_{0}$is the calendar year 2009 in which increase uptake of MC among youth is reported through year 2010 [[2](#_ENREF_2)] while $T_{0}$ is the year in which the intervention starts (2014). The values of the parameters $ç_{b}$and $ç_{v}$is set such that the fraction circumcised among newly tested HIV-uninfected uncircumcised young men reaches target coverage value (e.g. 37% in the fitted case and 50% or 80% in the intervention case). With 48% MC prevalence among young men before 2009, further MC uptake at 37% in the fitted case amounts to increasing the fraction of young men who are circumcised to 67%.

### 4. PrEP

Kenya has no reported significant historical levels of PrEP intervention, though there is now a goal of broader national rollout in 2017. Therefore, we assume PrEP starts in year $T_{0}$and females ($k$=3) of age 20-24 years old ($i$=5) who are at high risk of HIV, in categories ($p$=1, $o$=1), and newly diagnosed with HIV ($n$=2) are the target population. To define the fraction in category ($o$=1), we estimate the proportion of females (age 20-24) who can take PrEP at 1/5^th^ of girls, informed by the proportion who are not pregnant and not planning to be pregnant [[16](#_ENREF_16)].

PrEP intervention is implemented by moving susceptible females ($k$=3; $l$=1,2,3; $i$=5; $p$=1; $o$=1; $n$=2) to ($k$=3; $l$=1,2,3; $i$=5; $p$=2; $o$=1; $n$=2) using the adjustable parameters as in the following equation:

----------------(14)

where the parameters are adjusted by two parameters and $P_{l}$ assuming hierarchical structure (1:10:20) for the probabilities $P_{l}$ of finding a girl (age 20-24) in sexual activity groups $l$engaging in high-risk sex. The parameter is adjusted such that the resulting proportions of females recruited to PrEP in each risk activity ($f_{l}^{H}$) adds up to give the fraction of sexually active young women (age 20-24) who engage in high risk sexual intercourse [[16](#_ENREF_16)] ($\bar{r}_{fH}$):

----------------------(14)

We assume that girls on PrEP stop using PrEP immediately (return to category $p$=1) if they get infected and HIV-diagnosed ($n$=2,3) otherwise they stop PrEP whenever their age becomes ≥25 years old.

### 5. Cash transfer (CT)

The cash transfer (CT) is applied to females of age 15-19 starting from year $T_{0}$. Only females who are in category $o$=1 can be put on CT. To define the fraction in category ($o$=1), we estimate the proportion of females (age 15-19) who are in school at 1/5^th^ of girls [[16](#_ENREF_16)]. CT is implemented by moving newly tested HIV-uninfected females ($k$=3; $l$=1,2,3; $i$=4; $o$=1; $n$=2) to ($k$=4; $l$=1,2,3; $i$=4; $o$=1; $n$=2) using the adjustable parameters $\bar{\chi}_{klipno}$as in the following equation

----------------------(15)

Females leave CT and return immediately to category$k$=3 if they age older or they get infected and HIV-diagnosed ($n$=2,3). Females in category$k$=4 are characterized by reduced number of partnerships with older men who are 20+ years old. With the CT intervention the total number of partnerships per year formed by females need to be updated to accommodate changes in sexual mixing behaviors for category $k$=4 females in the following way:

$C_{kli}\to\bar{C}_{kli}=\left\{\begin{matrix} 0,t\geq T_{0},k=4,i=4 \\ unchanged,otherwise \end{matrix}\right.$ -----------------------(16)

With this change applied to equations (3), (7), and (8a), the parameter $\bar{\chi}$ controls the proportion ($\Xi$) of all partnerships formed by targeted girls that are with 20+ men given by:

----------------------(17)

The percentage reduction in $\Xi$ is the measure used for CT coverage in the main text.

### Model Inputs

| Table B: Model Assumptions |  |  |
| --- | --- | --- |
| Parameter | Default value | Sources |
| Demography and sexual behavior | | |
| Mortality rate | Table C |  |
| Fertility rate | Table C |  |
| Per capita growth rate | 0.045 |  |
| Cut-off age difference in partnerships between females and males | 10 years | [[17](#_ENREF_17)] |
| Proportion of population born into each sexual activity group |  |  |
| Number of unprotected sexual acts per partnership in a partnership: |  |  |
| With low sexual activity individual | 100 | Based on reported frequency of sex in marital relationships in sub-SAf countries [[18](#_ENREF_18)] |
| Otherwise | 2 |  |
| Proportion circumcised prior to sexual debut | 0.48 | Assumed to be flat over ages to calibrate model to circumcision prevalence reported in surveys [[15](#_ENREF_15)] |
| Proportion of in-school females (age 15-19); thus eligible for CCT intervention | 0.20 | Based on net attendance rates [[16](#_ENREF_16)] |
| Proportion of females (age 20-24) who can take PrEP | 0.20 | Estimated based on percentage not currently pregnant and not desiring more children [[16](#_ENREF_16)] |
| Proportion of sex acts protected adequately by condoms | Table D | Fitted |
| Balance parameter (extent to which sexual mixing is controlled by males sexual behavior) | 0.5 | Assuming equal role of men and women in the formation of partnerships |
| Mother to child HIV transmission probability | 0.3 | [[19](#_ENREF_19)] |
|  |  |  |
| HIV pathogenesis |  |  |
| Mean years in each stage of infection for untreated HIV |  |  |
| In stage of acute infection | 0.25 | [[20](#_ENREF_20)] |
| In the stages I and II of infection | Table E |  |
| In stage III of infection | 1.40 | Estimated |
| In stage IV of infection | 0.83 | Marks 10 months with heightened viremia [[20](#_ENREF_20)] |
| In the AIDS stage | 0.75 |  |
| Excessive mortality (when initiating ART at CD4>250) compared to non-infected persons | 5.0 per 100 person-years | [[21](#_ENREF_21)] |
| Excessive mortality (when initiating ART at CD4≤250) compared to non-infected persons | 8.0 per 100 person-years | Representative for heightened morbidity in patients receiving ART at CD4 <200 compared to patients starting ART at CD4>200 [[22](#_ENREF_22),[23](#_ENREF_23)] |
| Relative change in baseline HIV transmission probability |  |  |
| From population with acute infection | 26 | [[20](#_ENREF_20)] |
| To females | 2 | [[24](#_ENREF_24),[25](#_ENREF_25)] |
| To males | 1 (no change) | [[24](#_ENREF_24),[25](#_ENREF_25)] |
| From HIV infected individuals at risk of opportunistic infections and heightened viremia in late symptomatic infection stages | 7 | [[20](#_ENREF_20)] |
| From individuals with AIDS | 0 | Estimated based on the observation that sexual activity stops entirely ~1 year before death from infection [[26](#_ENREF_26),[27](#_ENREF_27)] |
| From individuals on ART | 0.08 (96% reduction) | ART efficacy of reducing onward transmission for persons on ART versus persons not on ART and in the asymptomatic stages of infection [[28](#_ENREF_28),[29](#_ENREF_29)] |
| Condoms both ways | 0.20 (80% reduction) | [[30](#_ENREF_30),[31](#_ENREF_31)] |
| Other parameters |  |  |
| Percentage reduction in susceptibility to HIV infection of circumcised male’s compared to uncircumcised males | 60% | [[32](#_ENREF_32),[33](#_ENREF_33),[34](#_ENREF_34)] |
| Dropout rate from treatment | 10.2 per 100 person-years | Based on data from AMPATH that 40% of those on ART drop out from treatment over 5 years period corresponding to the worst case reported over 2 years in [[35](#_ENREF_35)] |

Table C: Mortality (deaths per person-year) and fertility rates (births per female’s person-year by age. Sources ([[36](#_ENREF_36)], and assuming 36% reduction in under 5 mortality reported for Kenya)

| Age | Mortality for males | Mortality for females | Fertility for females |
| --- | --- | --- | --- |
| 0-4 | 0.127 | 0.127 | 0.000 |
| 5-9 | 0.007 | 0.007 | 0.000 |
| 10-14 | 0.007 | 0.007 | 0.000 |
| 15-19 | 0.002 | 0.002 | 0.120 |
| 20-24 | 0.003 | 0.004 | 0.270 |
| 25-29 | 0.004 | 0.005 | 0.240 |
| 30-34 | 0.005 | 0.006 | 0.210 |
| 35-39 | 0.006 | 0.005 | 0.120 |
| 40-44 | 0.010 | 0.007 | 0.060 |
| 45-49 | 0.010 | 0.007 | 0.017 |
| 50-54 | 0.014 | 0.010 | 0.000 |
| 55-59 | 0.019 | 0.014 | 0.000 |
| 60-64 | 0.027 | 0.020 | 0.000 |
| 65+ | 0.037 | 0.028 | 0.000 |

Table D: Fitted proportion of sex acts protected adequately by condoms for the study population of Nyanza, Kenya before the start of interventions (Sources [[36](#_ENREF_36)]). A factor reduction of 0.5 is assumed of these values when the partnership is between young females (age ≤24) and older males (age ≥30) as these partnerships in SSA are characterized by relatively lower condom use [[1](#_ENREF_1),[37](#_ENREF_37),[38](#_ENREF_38),[39](#_ENREF_39)].

|  | **Female activity group** | | |
| --- | --- | --- | --- |
| **Male activity group** | **Low** | **Intermediate** | **High** |
| **Low** | 0.0008 | 0.0008 | 0.16 |
| **Intermediate** | 0.0008 | 0.0008 | 0.16 |
| **High** | 0.16 | 0.16 | 0.57 |

Table E: Mean duration in the infection stages I and II (untreated HIV). Sources [[6](#_ENREF_6),[40](#_ENREF_40),[41](#_ENREF_41)]

| Age | Mean years spent in stage I | Mean years spent in stage II |
| --- | --- | --- |
| 0-4 | 0.04 | 0.04 |
| 5-9 | 5.6 | 5.6 |
| 10-14 | 5.6 | 5.6 |
| 15-19 | 6.1 | 5.0 |
| 20-24 | 5.9 | 4.8 |
| 25-29 | 5.2 | 4.3 |
| 30-34 | 4.2 | 3.5 |
| 35-39 | 3.7 | 3.0 |
| 40-44 | 3.3 | 2.7 |
| 45-49 | 2.2 | 1.8 |
| 50-54 | 1.7 | 1.4 |
| 55-59 | 1.1 | 0.9 |
| 60-64 | 1.1 | 0.9 |
| 65+ | 1.1 | 0.9 |

Table F: Estimated Mean number of partnerships formed per year based on fitting to age and gender-specific HIV prevalence for the study population of Nyanza, Kenya.

|  |  | **Women** |  |  |  | **Men** |  |
| --- | --- | --- | --- | --- | --- | --- | --- |
| **Age** | **Low** | **Intermediate** | **High** |  | **Low** | **Intermediate** | **High** |
| 10-14 | 0.111 | 0.975 | 12.906 |  | 0.015 | 0.136 | 1.802 |
| 15-19 | 0.292 | 2.570 | 34.011 |  | 0.025 | 0.218 | 2.883 |
| 20-24 | 0.865 | 7.623 | 100.91 |  | 0.986 | 8.688 | 115.00 |
| 25-29 | 0.616 | 5.431 | 71.887 |  | 2.467 | 21.740 | 287.76 |
| 30-34 | 0.454 | 4.002 | 52.967 |  | 2.872 | 25.312 | 335.04 |
| 35-49 | 0.002 | 0.019 | 0.248 |  | 0.007 | 0.060 | 0.792 |
| 50+[[1]](#footnote-1) | 0.828 | 7.295 | 96.556 |  | 1.108 | 9.768 | 129.30 |

Data on population growth rate, age stratified fertility and mortality rates are taken from the 1998 Kenya Demographic and Health Survey (KDHS) – i.e., before substantial AIDS-generated demographical changes had occurred and when no wide spread HIV prevention interventions existed. The model correctly predicts the population of Nyanza reported in census data after 1998 [[42](#_ENREF_42),[43](#_ENREF_43)](Figure B). The age-specific total survival duration of HIV infection (ART naïve population) is in Todd et al analysis [[6](#_ENREF_6)], which represents in its denominator a large variety of viral subtypes and of the genetic background of the study participants. Further, the probability of male-to-female HIV transmission is assumed to be twice that of female-to-male [[44](#_ENREF_44)].

Figure B: Model calibration to population size in Nyanza as reported in census data in 1999 and 2009 [[42](#_ENREF_42),[43](#_ENREF_43)].

# D. Cost Assumptions

Persons who are HIV-diagnosed (testing categories$n=2,3$) and have not yet initiated treatment are assumed to be in HIV care with appropriate CD4 monitoring and HIV care costs are accumulated for them.

| Table G: Disability weights for HIV health states (Source:[[45](#_ENREF_45)])   | Stage | Weight |  | | --- | --- | --- | | Uninfected | 0 |  | | Acute | 0.005 |  | | CD4>350 | 0.053 |  | | 250<CD4<350 | 0.221 |  | | CD4<250 | 0.221 |  | | AIDS | 0.547 |  | | HIV infection during ART | 0.053 |  | | Drop out from ART | 0.221 |  | |
| --- | --- | --- | --- | --- | --- | --- | --- | --- | --- | --- | --- | --- | --- | --- | --- | --- | --- | --- | --- | --- | --- | --- | --- | --- | --- | --- | --- |

# Additional Results

Figure C: The population attributable fractions (PAFtra) for HIV transmissions from index youth partners among HIV transmissions accumulated from the start of the epidemic in Nyanza (values between 2004-2014 were reported in the main text).

Figure D: The population attributable fractions (PAFacq) for HIV transmissions to susceptible youth partners among HIV transmissions accumulated from the start of the epidemic in Nyanza (values between 2004-2014 were reported in the main text).


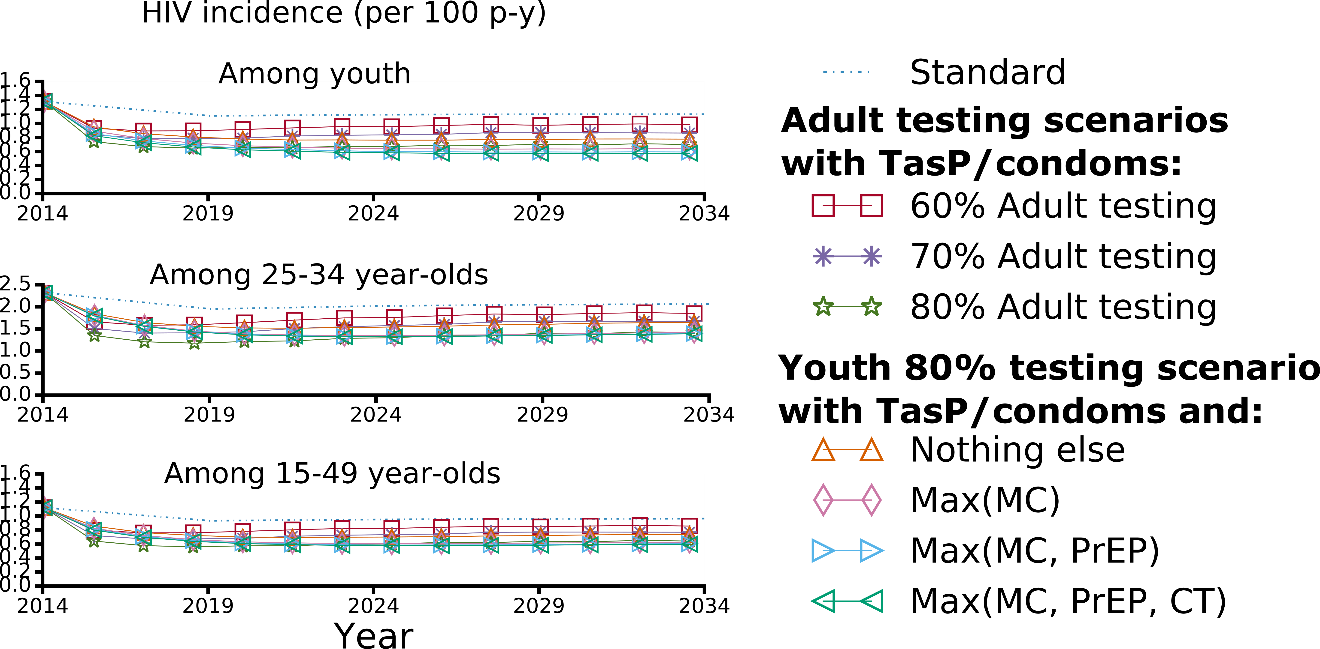
Figure E: Model HIV incidence by scenario among young persons (age 15-24) and their future partners (age 25-34) as well as the whole adult population (age 15-49). The standard scenario consists of 40% adult testing with TasP.


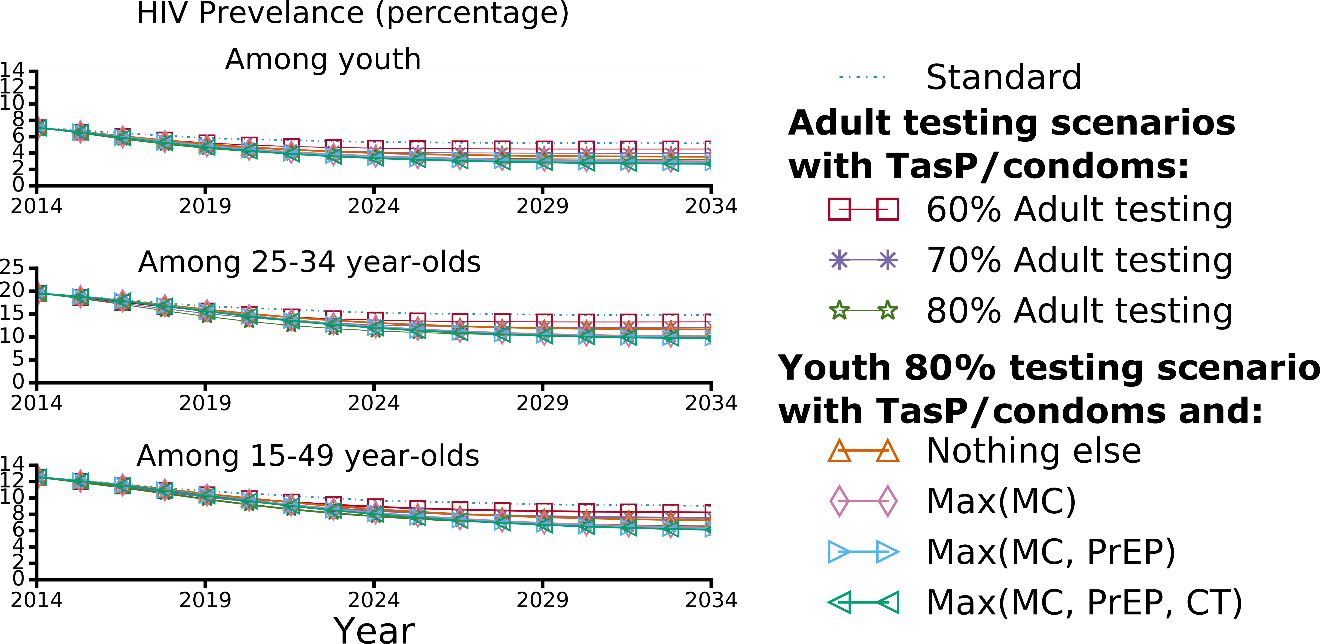
Figure F: Model HIV prevalence by scenario among young persons (age 15-24) and their future partners (age 25-34) as well as the whole adult population (age 15-49).


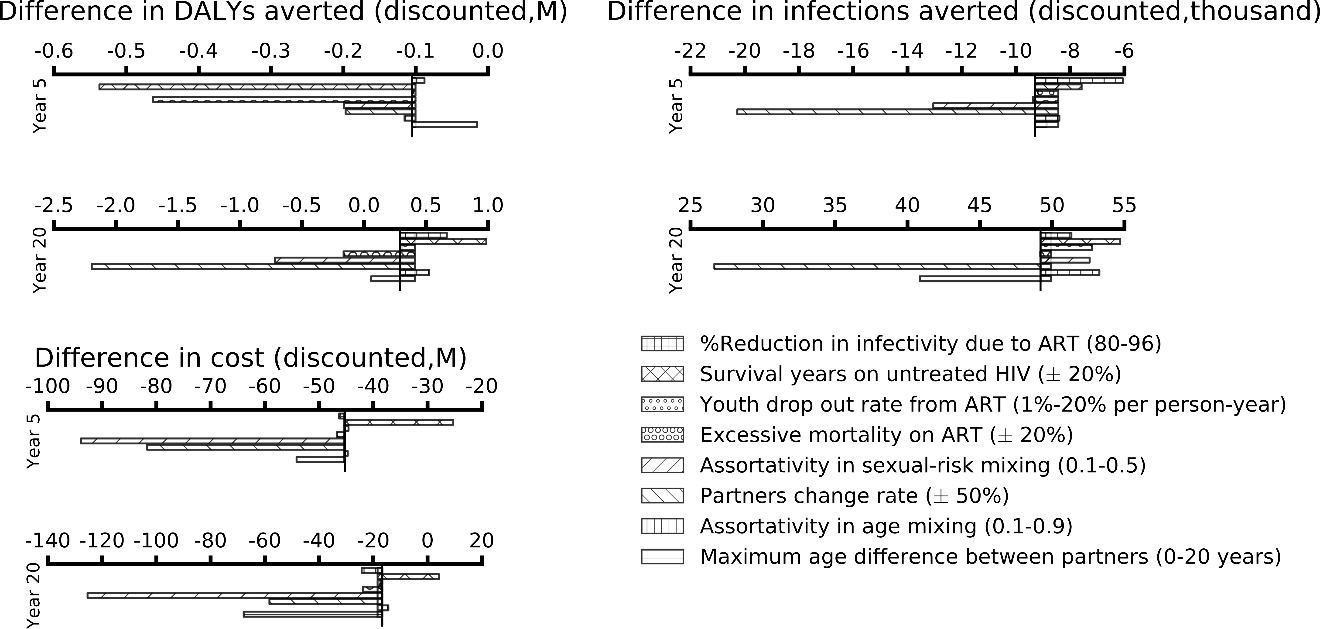


Figure G: One-way sensitivity analyses assessing the robustness of model predictions in year 5 and year 20 on the differences in DAYLs averted, infections averted and costs between two interventions to uncertainties in specifying model parameters. The two scenarios/strategies are (1) a youth-focused 80% HIV testing with TasP/condoms and 80% MC uptake among HIV-negative young men and (2) an adult-focused 70% HIV testing with TasP/condoms. Variation in the predicted differences of (value of outcome under the youth-focused package minus value of outcome under the adult-focused package) are shown. M=million.

.

# References

1. Ghys PD, Gouws E, Lyerla R, Garcia Calleja JM, Barrerre B, et al. (2010) Trends in HIV prevalence and sexual behaviour among young people aged 15-24 years in countries most affected by HIV. Sex Transm Infect 86 Suppl 2: ii72-ii83.

2. NACC (2010) United Nations Genral Assembly Special Session on HIV and AIDS (UNGASS), Country Report - Kenya.

3. NASCOP (2005) Kenya National AIDS and STI Control Program (NASCOP), Ministry of Health. Kenya. AIDS in Kenya. Nairobi.

4. Calves, Anne-Emmanuele, Gretchen T. Cornwell, Enyegue. PE-d (1996) Adolescent Sexual Activity in Sub-SaharanAf- rica: Do Men Have the Same Strategies and Motivations as Women? University Park, PA: Population Research Institute.

5. Barker GK, Rich S (1992) Influences on adolescent sexuality in Nigeria and Kenya: findings from recent focus-group discussions. Stud Fam Plann 23: 199-210.

6. Todd J, Glynn JR, Marston M, Lutalo T, Biraro S, et al. (2007) Time from HIV seroconversion to death: a collaborative analysis of eight studies in six low and middle-income countries before highly active antiretroviral therapy. AIDS 21 Suppl 6: S55-63.

7. Kenya (1993) National Council for Population and Development (NCPD), Central Bureau of Statistics (CBS) (Office of the Vice President and Ministry of Planning and National Development [Kenya]), and Macro International Inc (MI). 1994. Kenya Demographic and Health Survey (KDHS) 1993. Calverton, Maryland.

8. Cheluget B, Baltazar G, Orege P, Ibrahim M, Marum LH, et al. (2006) Evidence for population level declines in adult HIV prevalence in Kenya. Sexually transmitted infections 82 Suppl 1: i21-26.

9. Kenya (1998) National Council for Population and Development (NCPD), Central Bureau of Statistics (CBS) (Office of the Vice President and Ministry of Planning and National Development [Kenya]), and Macro International Inc (MI). 1999. Kenya Demographic and Health Survey (KDHS) 1998. Calverton, Maryland.

10. Schoub BD, Smith AN, Lyons SF, Johnson S, Martin DJ, et al. (1988) Epidemiological considerations of the present status and future growth of the acquired immunodeficiency syndrome epidemic in South Africa. S Afr Med J 74: 153-157.

11. Karcher H, Omondi A, Odera J, Kunz A, Harms G (2007) Risk factors for treatment denial and loss to follow-up in an antiretroviral treatment cohort in Kenya. Trop Med Int Health 12: 687-694.

12. NASCOP (2009) Kenya National AIDS and STI Control Program (NASCOP) Department of Strategic Information, Surveillance and Research. Draft of Annual Health Sector HIV Report. Nairobi.

13. NASCOP (2012) Personal Communication: Persons started on ARVs by WHO stages in 2011.

14. Baveewo S, Ssali F, Karamagi C, Kalyango JN, Hahn JA, et al. (2011) Validation of World Health Organisation HIV/AIDS clinical staging in predicting initiation of antiretroviral therapy and clinical predictors of low CD4 cell count in Uganda. PLoS One 6: e19089.

15. KAIS (2007) Kenya AIDS Indicator Survey Nairobi, Kenya.

16. Kenya (2009) National Bureau of Statistics (KNBS) and ICF Macro 2010. Kenya Demographic and Health Survey (KDHS) 2008-09. Calverton, Maryland.

17. Ott MQ, Barnighausen T, Tanser F, Lurie MN, Newell ML (2011) Age-gaps in sexual partnerships: seeing beyond 'sugar daddies'. AIDS 25: 861-863.

18. Johnson LF, Dorrington RE, Bradshaw D, Wyk VP-V, Rehle TM (2009) Sexual behaviour patterns in South Africa and their association with the spread of HIV: Insights from a mathematical model. Demographic Research 21: 289-340.

19. Dabis F, Ekpini ER (2002) HIV-1/AIDS and maternal and child health in Africa. Lancet 359: 2097-2104.

20. Bezemer D, de Wolf F, Boerlijst MC, van Sighem A, Hollingsworth TD, et al. (2008) A resurgent HIV-1 epidemic among men who have sex with men in the era of potent antiretroviral therapy. AIDS 22: 1071-1077.

21. Brinkhof MW, Boulle A, Weigel R, Messou E, Mathers C, et al. (2009) Mortality of HIV-infected patients starting antiretroviral therapy in sub-Saharan Africa: comparison with HIV-unrelated mortality. PLoS Med 6: e1000066.

22. Lawn SD, Bekker LG, Myer L, Orrell C, Wood R (2005) Cryptococcocal immune reconstitution disease: a major cause of early mortality in a South African antiretroviral programme. AIDS 19: 2050-2052.

23. Lawn SD, Myer L, Bekker LG, Wood R (2006) Burden of tuberculosis in an antiretroviral treatment programme in sub-Saharan Africa: impact on treatment outcomes and implications for tuberculosis control. AIDS 20: 1605-1612.

24. Padian NS, Shiboski SC, Jewell NP (1991) Female-to-male transmission of human immunodeficiency virus. JAMA 266: 1664-1667.

25. Nicolosi A, Musicco M, Saracco A, Lazzarin A (1994) Risk factors for woman-to-man sexual transmission of the human immunodeficiency virus. Italian Study Group on HIV Heterosexual Transmission. J Acquir Immune Defic Syndr 7: 296-300.

26. Quinn TC, Wawer MJ, Sewankambo N, Serwadda D, Li C, et al. (2000) Viral load and heterosexual transmission of human immunodeficiency virus type 1. Rakai Project Study Group. N Engl J Med 342: 921-929.

27. Pilcher CD, Joaki G, Hoffman IF, Martinson FE, Mapanje C, et al. (2007) Amplified transmission of HIV-1: comparison of HIV-1 concentrations in semen and blood during acute and chronic infection. AIDS 21: 1723-1730.

28. Donnell D, Baeten JM, Kiarie J, Thomas KK, Stevens W, et al. (2010) Heterosexual HIV-1 transmission after initiation of antiretroviral therapy: a prospective cohort analysis. Lancet 375: 2092-2098.

29. Cohen MS, Chen YQ, McCauley M, Gamble T, Hosseinipour MC, et al. (2011) Prevention of HIV-1 Infection with Early Antiretroviral Therapy. N Engl J Med.

30. Hughes JP, Baeten JM, Lingappa JR, Magaret AS, Wald A, et al. (2012) Determinants of per-coital-act HIV-1 infectivity among African HIV-1-serodiscordant couples. J Infect Dis 205: 358-365.

31. Weller S, Davis K (2002) Condom effectiveness in reducing heterosexual HIV transmission. Cochrane Database Syst Rev: CD003255.

32. Auvert B, Taljaard D, Lagarde E, Sobngwi-Tambekou J, Sitta R, et al. (2005) Randomized, controlled intervention trial of male circumcision for reduction of HIV infection risk: the ANRS 1265 Trial. PLoS Med 2: e298.

33. Gray RH, Kigozi G, Serwadda D, Makumbi F, Watya S, et al. (2007) Male circumcision for HIV prevention in men in Rakai, Uganda: a randomised trial. The Lancet 369: 657-666.

34. Bailey RC, Moses S, Parker CB, Agot K, Maclean I, et al. (2007) Male circumcision for HIV prevention in young men in Kisumu, Kenya: a randomised controlled trial. The Lancet 369: 643-656.

35. Rosen S, Fox MP, Gill CJ (2007) Patient retention in antiretroviral therapy programs in sub-Saharan Africa: a systematic review. PLoS Med 4: e298.

36. Kenya (1989) National Council for Population and Development and IRD Macro Systems, Inc. 1989. Kenya Demographic and Health Survey (KDHS) 1989. Columbia, Maryland.

37. Report (2003) Zimbabwe Human Development Report 2003 Redirecting our responses to HIV and AIDS.Harare: UNDP/Institute Development Studies.

38. Luke N, Kurz KM (2002) AIDSMark project: Cross-generational and Transactional Sexual Relations in Sub-Saharan Africa: Prevalence of Behavior and Implications for Negotiating Safer Sexual Practices. USA: ICRW and PSI

39. Gouws E (2010) Trends in HIV prevalence and sexual behaviour among young people aged 15-24 years in countries most affected by HIV. XVIII International AIDS Conference. Vienna, Austria.

40. WHO (2010) Toward universal access: Scaling up priority HIV/AIDS interventions in the health sector (available : <http://www.who.int/hiv/pub/2010progressreport/en/)>. Accessed on April 18, 2013.

41. Wandel S, Egger M, Rangsin R, Nelson KE, Costello C, et al. (2008) Duration from seroconversion to eligibility for antiretroviral therapy and from ART eligibility to death in adult HIV-infected patients from low and middle-income countries: collaborative analysis of prospective studies. Sex Transm Infect 84 Suppl 1: i31-i36.

42. Kenya (1999) National Bureau of Statistics (KNBS). Kenya Population and Housing Census 1999. Available (<http://www.knbs.or.ke/censusdocuments.php)>. Accessed on 05/23/2012.

43. Kenya (2009) National Bureau of Statistics (KNBS). Kenya Population and Housing Census 2009. Available (<http://www.knbs.or.ke/censusdocuments.php)>. Accessed on 05/23/2012.

44. Gray RH, Wawer MJ, Brookmeyer R, Sewankambo NK, Serwadda D, et al. (2001) Probability of HIV-1 transmission per coital act in monogamous, heterosexual, HIV-1-discordant couples in Rakai, Uganda. Lancet 357: 1149-1153.

45. Salomon JA, Vos T, Hogan DR, Gagnon M, Naghavi M, et al. (2012) Common values in assessing health outcomes from disease and injury: disability weights measurement study for the Global Burden of Disease Study 2010. Lancet 380: 2129-2143.

1. Model baseline characterizations are not highly sensitive to sexual activity in this age category [↑](#footnote-ref-1)
